# Paralysis and Convulsions as Effects of Diseases of the Base of the Brain

**Published:** 1878-05

**Authors:** 


### Chicago Medical Journal

AND

### EXAMINER.

Vol. XXXVI.—MAY, 1878.—No. 5.

### Original Lectures.

### PARALYSIS AND CONVULSIONS AS EFFECTS OF DISEASES OF THE BASE OF THE BRAIN.

Delivered in Chicago, February 21st, 22d and 23d, 1878.

By Dr. Brown-Séquard.

*(Reported for the Journal and Examiner.)*

THIRD LECTURE.

Gentlemen: I have to-day, to treat at length, or at least, as far as time will allow, of three distinct points.

First, the basis that I can offer for the new views of paralysis and convulsions, caused by disease of the brain.

I have next to examine the diagnosis of disease of the brain cells from the new point of view.

In the third place, I have also to give the conclusions that can be drawn from the facts given in the two preceding lectures, as regards treatment.

And first, I have to speak of paralysis, and to examine the possible support of the views I have expressed. What I maintain is, that paralysis appears in case there is a destruction of tissue in the part of the brain where there is disease, and that the consequent irritation of the cells of grey matter distributed in many parts of the brain, or in only a few parts, or perhaps also (and most likely), in some parts of the spinal cord, there acts on these cells so as to arrest their activity. This view, as you can easily understand, is very radical, and it was not without some hesitation that I put it forward for the first time a few years ago. But since then, facts have come to light which seem to give a clear and decided emphasis to these views.

On the other hand, the necessity of replacing the views which I think ought to be abandoned altogether, and which are now generally admitted, requires that for a time, at least, till we know better, we should have some theory to connect the facts and serve to promote further progress. You well know that in science, a theory which is in harmony with every fact that is known or developed, and not in contradiction with any, ought (if there is none better) to be accepted, at least temporarily. It may prove to be true, and it may prove to be completely the reverse; but for the time being, it ought to be accepted, because we need a theory for the development of our knowledge. Therefore, till I know that there are facts in opposition to the views that I have expressed, I think it is essential that these views be admitted at least by myself first, and if any one among my hearers feels inclined to accept these views, I believe they may serve till something better is found.

We find sometimes that an affection of the brain, of minute extent, having existed there for some time, but having given no manifestation whatever, will suddenly produce a paralysis. As we find, in the autopsy, that the lesion is very limited, and that there is disorganization of but a small part of the brain, we certainly must admit that something has irritated an organ, and thus produced paralysis. Otherwise, we could not understand how such a minute lesion could produce such a complete general paralysis. Now, put such a fact in the presence of another, such for example as I once observed in London. A patient was brought to me, as one attacked with general paralysis, and having certainly the characteristics of that affection; I detected (when he was leaving my office, where I had prescribed as if it was a pure case of paralysis) that when his hand was brought to a certain organ, the irritation was considerable at that part; and on examination, I found that balanitis existed. Noting this, I examined further into the case, and found symptoms of paralysis of the brain. The symptoms were slight, and they were covered and crowded out by other symptoms. The treatment appropriate to that affection, the inflammation of the prepuce, was applied, and in less than a week the patient was radically cured. In that case there was external irritation of no considerable extent, and acting through the organ which has great power in certain individuals, on the brain. In that case we had a general paralysis, and if slight irritation can produce a slight paralysis, considerable irritation should produce greater paralysis.

I could mention a great many facts which show that in animals we can produce paralysis by slight irritation. I have already spoken of paralysis that consists of a loss of the action of muscles and blood vessels on one side, together with paralysis of sensibility in the opposite side, and shown that this may come from a prick of the posterior column of the spinal cord as well as section of the cord. It is not certain that destruction of tissue always produces paralysis; but it certainly comes through an influence exerted by irritation propagated through the cells so as to cause paralysis and other symptoms. It is the same in many of these cases in which, by affecting the sciatic nerve in animals, I have caused epilepsy, and have seen frequently after a division of the sciatic nerve, more or less paralysis of the abdomen and spine, and in each lower part on the same side. There is also, not always but in many instances, paralysis of the muscles above the place of section of the sciatic nerve, which receive no fibers from other nerves than the sciatic. But the most clear series of facts showing that we can produce paralysis by the mere irritation of certain nerves and certain parts of the nervous centers, is that in which, by a mere prick of certain parts of the *medulla oblongata*, symptoms of general and complete paralysis are produced, with loss of consciousness. The animal falls, as it were dead from the prick. The action of the heart is weakened; the breathing ceases completely. There is no appearance of life so long as you do not excite the skin, by means of which you can produce reflex action, showing that the reflex power of the cord is not lost; but the power of the will is lost, and if you lift the limb it drops with complete loss of consciousness; the heart ceases to beat and death comes. There are many other features most interesting indeed, but which I have not time to dwell upon now. I will only say that changes take place to such an extent in the blood and other parts, that putrefaction may be delayed from 30 to 50 days after death, and that the body may be left in a room at a temperature of 70° for a long time without decomposition. Such facts show that not only paralysis may appear, but other phenomena from the arrest of the activity of the cells. If we study what takes place in man under similar circumstances, we find that phenomena more or less like these will ensue. There are instances where a single wound in a more sensitive part of the body, as in the hand or foot, has produced similar effects. In one case I saw such effects result from a mere prick. The patient was to be bled; the lancet had not gone in before the patient fainted away, the heart ceased to beat, and the limbs seemed to be dead. The patient was recalled to life with the greatest difficulty. Death would have occurred, if the most energetic means had not been employed immediately. So that paralysis, or this cessation of activity of the cells employed by the will, can ensue from causes in appearance trifling. These effects certainly render quite forcible the theory I have put forward; and what remains to be explained is this: Why is it that paralysis will appear in one part of the body with a given lesion in one person, and in another part of the body with the same lesion in another person? I have already stated that, according to the conditions of excitability of the various parts of the body, the effects of irritation starting from a certain part, will be extremely various, and in this we have the great cause of the difference between the effects in different individuals produced by a similar lesion.

As regards other facts which are in favor of the view which I have put forward, you will notice, if it is a case of disease of the brain, that the heart's action will be diminished considerably. There we have a clear case of inhibition or arrest of activity of certain nerve cells; we have a clear proof that the phenomena of arrest, or inhibition if you like to call it so, will come from disease of the brain and proceed for days and days ; and there are cases on record in which, for many months, the pulse rate per minute has been 30 or 40. We see therefore that, together with the propagation of paralysis in these cases, there exists the clear influence of arrest, which is similar to the propagation of paralysis itself. Thc objection to the view I have put forward, which would be based upon the persistence of paralysis after the lesion of the brain is produced, may be answered. In putting together what relates to the heart and what relates to paralysis, this objection falls to the ground; as we know that it is from arrest that the heart's action is stopped or considerably diminished. And if that phenomenon can persist for days, weeks and months from a certain cause, we can expect also that the same thing will take place from influences connected with cells in the brain or spinal cord.

But there are other arguments more fully in favor of the views which I have proposed. Every physician here, every student knows that two kinds of paralysis may appear in cases of brain disease, and no doubt may appear very frequently, when the disease is in the base of the brain especially. Those two effects consist in paralysis of the sphincter of the bladder and of the sphincters of the rectum. These sphincters may lose their action altogether. Every one knows that these sphincters are not kept active by the brain. They are kept active by the cells in the gray matter of the spinal cord. It is clear, therefore, that the arrest of action of those muscular fibres which pass to the two sphincters, must be due to arrest of activity of the cells which keep them in a normal state of tension. In these cases, therefore, we have clear proof that disease of the brain can produce arrest of activity of cells in the spinal cord, just as it can produce it in the nerve cells of the heart. Therefore we have something similar to what I believe to exist in the arrest of activity of cells in the brain and spinal cord, producing paralysis.

There is another fact of great importance and of some purport, which is that the reflex faculty of the spinal cord may be lost completely in cases of disease of the brain. There we have clear proof that the cells in the spinal cord, which are used no doubt with reflex power, lose their activity; and the influence which comes from the brain must come in the same way as that which arrests the activity of the cells of the heart. We have, therefore, a number of facts showing that cells at a great distance from the brain—cells in the lower part of the spinal cord, as well as cells in the tissue of the heart—can lose their activity, can have their activity inhibited, as it is called, from an influence coming from the place where there is a disease of the brain. If so, we have something quite similar to what I suppose to exist in the propagation of paralysis. We have also in these facts which may be said at present to be numerous (they are very rare indeed, but I have collected a pretty large number of them, so that I am led to employ the expression "numerous"), we have facts, I say, which show that paralysis due to organic disease of the brain has disappeared more or less rapidly, and sometimes suddenly, the disease producing them still existing. In these cases, if we were not to admit that the paralysis was due simply to arrest of activity, we could not explain the phenomena. The array of facts is very large, in which it appears that a cessation of activity can be produced by mere irritation, and can disappear more or less suddenly, when the lesion, if it is a large one, persists.

Let us take what is known of amaurosis. A prick may produce amaurosis in some cases. On the other hand, we find in man, that amaurosis though appearing gradually and slowly in certain cases, has appeared suddenly, and in a few cases has disappeared as suddenly. The autopsy being made, however, the disease which produced the phenomena was still found to exist. Therefore we may admit that the activity of the cells in any part of the system may be stopped, may be arrested, may be inhibited for a time (it may be a very long time, or may be merely temporary) and be caused by an irritation in certain parts of the brain or other parts of the nervous system.

What now have we to say of the production of convulsions in cases of disease of the base of the brain ? Convulsions in these cases have certainly something peculiar in their production. Indeed, in the study of the effects, either of the common forms of convulsions, chronic movements, epilepsy, rigidity, trembling or any kind of involuntary movement, when these disordered movements appear to result from disease in any part of the base of the brain, we certainly find that the old views cannot be applied to explain the phenomena in a number of these cases. That we find irritation limited to a small number of fibers producing convulsions in the two sides of the body, is certainly true. We find that these kinds of disordered movements appeared in a great many cases on the side corresponding to that of the lesion. Whether the lesion is of the *crura cerebri*, or the *pons varolii*, we have something in complete discordance with what is admitted.

What about convulsions in these cases? As I have said, convulsions appear where motor fibers or motor centres are irritated, and the irritation is propagated by the cells of the nerves, acting upon this excitability so as to produce the phenomena of convulsions. If the cells of one part of the spinal cord or *medulla oblongata*, are more excitable than the cells of the opposite side, there will be convulsions; if the reverse, the convulsions will be on the other side. Indeed, I do not see that there can be any difficulty if you have this key or explanation, which consists in admitting that an irritation, starting from the place of the disease, proceeds to the parts so as to produce a morbid activity. If the cells are endowed with a power to produce movements, they produce convulsive movements, of one kind or another. We have, therefore, a common element, a common principle, which we may apply to the protection of these centers. In the two instances, according to the theory I propose, the irritation starts from a part where the disease is, and is propagated to the cells all about the system, and it may produce the one or the other effect. There is a similarity so far in the production of convulsions and paralysis in this respect, that in the two instances there is a common element of irritation starting from the place of the disease, and reaching the cells. When the cells are reached, then the variety may begin. If these cells are in a certain degree of excitability, which makes them lose the power of action, paralysis appears. If these cells, on the contrary, are excited like the cells of the spinal cord or *medulla oblongata*, and reflex movement takes place, the reflex movement occurs convulsively—either convulsion or stiffness or any kind of involuntary muscular contraction. It is exceedingly easy to explain the phenomena in that way, and I do not know of any facts in opposition to these views. If you do, I should like to hear of them.

There is, indeed, one point, which I mentioned in the beginning of this lecture, which relates to the diagnosis of convulsions and paralysis, when their cause is in the base of the brain. It may seem to some of you that the new views I propose render diagnosis more difficult than before. But it is certainly not so. Indeed you can easily understand *a priori* that if the other views were wrong, they could not help the diagnosis; they must have been the cause of mistakes. It will be certainly a good test of these new views if we find the diagnosis become easier for those who admit them. Upon this point I will say that I have been able in some cases to form a diagnosis, which would have been a matter of extreme difficulty if the old views had been admitted. If we find, for instance, a series of symptoms, such as a slight paralysis coming on secretly and slowly, perhaps in the right side of the body, an arm or a leg, and, together with the paralysis, a very slow anæsthesia and some slight degree of rigidity, we are led to think there is disease on the opposite side of the brain. But if we find the face paralyzed in its motor action on the same side, if we find that the susceptibility of the face is diminished on the same side, if we find the eye is inflamed, we have then together with the other facts I have mentioned, and with those I have not mentioned, a very great probability that a disease exists at the base of the brain, which affects the trigeminal nerve and produces the effects which we know appear when we divide that nerve. If, besides these symptoms, there is also some deafness on the same side, and if there is some strabismus, showing that the sixth pair of nerves is paralyzed so that the eye is drawn inward, that is another reason for believing that there is disease affecting the facial and the seventh, fifth and sixth pairs of nerves. If these nerves are diseased on the same side, and the paralysis in the limbs exist on the same side, you must either admit that there are two lesions in that man, or, if you are ready to admit that the paralysis can exist on the corresponding side of the lesion, then you are ready to admit that one single lesion can produce all these effects. How can you be sure as regards this? By the complete absence of symptoms of any kind in a case such as that

I have mentioned, when the paralysis is on the right side. You may certainly admit that there are two lesions, as that would be in harmony with the facts. You might admit a lesion of the nerves I have mentioned, without affection of the base of the brain. But if the paralysis has appeared at the same time that the symptoms appeared in the nerves I have mentioned, and if the paralysis have progressed *pari passu* in the same ratio of increase, then it is certainly evident that there is only one lesion. Thus you can arrive at the diagnosis by admitting the views I have set forth. It is only admitting that paralysis may be produced by disease in the base of the brain, on the corresponding side, which is altogether in opposition to the views generally admitted.

I have not time to insist upon this point, but will say that, as regards the diagnosis of the disease in the base of the brain, physiology, such as we know it now, may serve to help the diagnosis immensely. Indeed, when the lesion is in any part of the base of the brain which contains the nerves of the third pair and last cranial nerve, originating from the *medulla oblongata*, if you state with the greatest care all the symptoms that may appear in the eye, ear, face, tongue or throat, you find the seat of the disease pretty readily; but if you are inclined to admit that convulsions can appear on the same side and paralysis on the same side; if you are ready to admit that the four limbs are convulsed from a lesion limited to one side of the base of the brain; and if you put these facts together with the facts which show what is the seat of the disease, then you can localize the disease—you can diagnosticate the seat of the disease pretty readily. But if you admit the old theories, you are at sea and cannot come at a diagnosis in those cases in which the symptoms of paralysis or convulsions are not in harmony with the admitted theories; so that with these new views diagnosis may be aided rather than hindered.

I now come to the last part of this lecture, and that is, what rule of treatment can we draw from these new views, or from the facts on which these views are grounded? I should like to have more time to deal with this topic. But first, I will say that I believe a new field of therapeutics has been opened, and one which promises to be of the greatest advantage, by the acquisitions of science within the last 30 or 40 years. If we take, for instance, a case of convulsions, it clearly proves what I wish to explain. If we suppose, for instance, a case of disease in the dorsal part of the spinal cord, leaving a great part of the cord perfectly healthy, in such a case, as you well know, there are powerful convulsions. In such a case, simply treading on the big toe will stop the convulsions immediately. And by what mechanism is the rigidity stopped ? It is quite clear that in such cases there is a morbid activity of certain cells, which are continually throwing off nerve force. If an irritation comes from the big toe, it stops that activity. It is similar to what takes place when we stop the action of the heart by galvanizing the pneumogastric nerve. I have no knowledge whatever that can explain why this is so; but I do know that certain parts of the body are endowed with powers which other parts do not possess ; that an irritation in the sole of the foot, for instance, can produce reflex phenomena more powerfully than in other parts of the body. No doubt, in such cases, we have clear evidence that morbid activity of the cells can be stopped by mere local irritation. The same thing takes place in many cases of epilepsy. Muscular contraction, preceding the attack, and preceding the loss of consciousness, occurs. In many of these cases it is exceedingly easy to put an end to the attack, and to the loss of consciousness. Suppose it is the head that is first attacked ; suppose the head is brought with great force towards one shoulder. In such case, if the head is taken hold of firmly, and brought strongly towards the opposite shoulder, the attack will cease, certainly in 19 cases out of 20, if the patient has not lost consciousness; but it is essential that the consciousness be not lost; for when it is lost, and the attack is on, I do not know of any means that can make it cease.

There are means of diminishing the force of the attack, such as pressure on the pneumogastric or the neck. It was thought that pressure exerted on the carotid artery, reducing the amount of blood flowing to the brain, was beneficial. That may have a little truth in it. But the greatest effect which comes from the cessation of the attack after it has existed, is produced through the influence exerted on the heart, by pressure on the *par vagum.* Convulsions, therefore, may be prevented, and a loss of consciousness, and a complete attack of epilepsy may be prevented, by the mere action of certain nerves. When I first had a large field of practice in 1860, in London, I found in a number of cases in which there was muscular contraction and convulsions, that drawing on the muscles which contracted so as to elongate them, was generally sufficient to stop the convulsions; but I found also that it was not quite essential to do that, in many cases a mere bandaging of the skin was quite enough. In many cases the application of a ligature is sufficient. It is not because the ligature prevents something from passing, as was formerly imagined; but, on the contrary, because the ligature sends something to the brain. It changes the activity and stops the attack. So true is this, that the best plan consists in not applying a ligature in the ordinary way, but in applying a series of ligatures like handkerchiefs, and drawing them again and again over the surface, so as to irritate the skin. The ligature is not essential. All that is necessary is merely to bandage the part or to apply over it the cautery, or a piece of ice, or anything that can irritate the skin considerably and rapidly. It is quite clear, therefore, that when we stop an attack of epilepsy in this way, we produce an irritation, which irritation goes to the base of the brain, and there stops the activity of the cells, which morbid activity was preparing an attack of epilepsy.

As regards these phenomena of arrest, these inhibitory phenomena may be employed to prevent attacks of hysteria; also a good many other kinds of attack, such as catalepsy, or other convulsive phenomena of various names and forms. In these the irritation of certain parts can produce a cessation of the attack. As regards hysteria, pressure on the ovaries will increase the hysterical phenomena; but in some attacks of convulsions, pressure on the ovaries will stop an attack.

The other means of treatment which I have employed with great success, consists in raising the two arms of the patient over the head suddenly. That is quite sufficient sometimes. Any great irritation can succeed. But if you find a patient on whom the exercise of irritation is absolutely useless, do not give up the attempt, but adopt other means of counter-irritation, and you will have a great chance of being rewarded. It is most important to preserve the patient from an attack, not because you save him from that attack merely, but because any attack of a nervous affection is the cause of after attacks, owing to the change in the circulation which takes place in the base of the brain. This is especially the case with epilepsy. Saving the patient from an attack of epilepsy is saving him from a series of such attacks. These views as regards the arrest of activity of the cells, so far as epilepsy is concerned, find confirmation in certain effects which have been observed as regards paralysis or other forms of cessation of activity of cells, as in hysteria.

Consider what relates to anæsthesia. For instance, a friend of mine found that the passage of the galvanic current in cases of anæsthesia would cause the anæsthesia to cease. Thus we have a proof that anæsthesia, although due to organic disease in some cases, and, as all theories have it, due also to destruction of tissue in part, yet there remains the power to perceive sensation in some degree, or we could not cure it so rapidly. In researches made by Prof. Vulpian, other cases have been found. The galvanic current has cured amaurosis. No other way of explaining these cures can be found except this, that amaurosis, as well as anæsthesia, is due to the arrest of activity of cells, and that the irritation which the galvanism produces, causes, in its turn, the highest activity of cells in the part where the disease is. This morbid activity of some cells is thus stopped by the activity of other cells. If the part where the disease exists is acted upon by the galvanic current, so that the cells lose their activity, then the bad effect ceases. We have a cessation of activity of certain cells in a morbid state, and, as a consequence of that cessation of activity, we obtain a return of the normal state of the cells, which were kept in abeyance by the action of the cells where the disease exists. Whatever this theory is worth, the facts are most interesting.

Since these facts have been published, others have been brought to the attention of the medical public, which had been studied with great care by Burke in Paris, but which had been rejected or which had not attracted attention. The other facts, since Prof. Charcot took them up, have become an important field of study. Prof. Charcot took the facts of Burke—facts showing that the application of metallic plates on the skin have been sufficient to cure anæsthesia, and has shown that pretty much what was obtained by Vulpian by the galvanic current, can be produced by plates of metal—as a piece of copper or other metal. There are some patients who will be affected by gold, who will not be affected by copper; so that if you try experiments with these metals, you ought to try alternately gold and copper.

In facts of this kind it is quite clear that if we stop anæsthesia, it must be from the arrest of the morbid activity of certain cells. Now, what has been observed in these cases? As regards anæsthesia, as has been observed, there are other means of treatment, and the field has not yet been fully explored; but some of the facts which I have presented, and which are becoming more and more numerous, lead us to admit that in cases of paralysis due to brain disease, or disease of the spinal cord, the best mode of treatment by counter irritation consists in applying counter irritation over the spine or nape of the neck, but chiefly in applying the counter irritation to the limbs and especially the paralyzed ones—not exclusively to these limbs but chiefly there. I should say that Dr. Graves of Dublin, after having tried these means, adopted the theory that in cases of disease of the spinal cord, counter irritation ought not to be applied to the spine, but to the lower limbs. But I cannot accept his theory. When I took up the subject, I began in cases of locomotor ataxy, and applied counter irritation to the limbs, and I found that the means had the power to diminish the disease in a number of cases, especially when the disease had not extended to the grey matter and was not accompanied by paralysis of the centers, but only by ataxic movement. I applied a plaster to the limb, all around, about an inch and a half or two inches in width. If on the thigh, it must be only about one inch or one inch and a quarter. As you move down the leg, the shorter the circumference of the limb, the broader the band of the plaster should be. This circular plaster has an immense power. In the two cases where organic disease produces paralysis and when epilepsy exists without organic disease, pretty much the same condition of things exists. It is a change in the activity of the cells in the base of the brain or the spinal cord, which is produced by this peripheric irritation. There is no doubt that all sorts of means of irritation have more power in these cases applied over the limbs and especially those which are paralyzed, than when applied to the head, neck or spine in any part of its length. Still you must not lose sight of the fact that from the *vaso-motor* nervous system of the head, arises the spinal cord on a level with the second or third vertebra, and then when you wish to produce a change in the circulation, you must apply counter irritation; and among the means of counter irritation most powerful to produce change, is ice applied over the skin—not in a bag. The bag is too thick. Anything interposed between the ice and the skin, diminishes its action. Ice applied four to eight minutes on the bare skin, is a means of great power.

Next to that the cautery, applied so as to produce a simple drying of the skin, is a good means. And you obtain this without giving rise to pain, or without having its application followed by pain. You obtain this result by the use of a pointed instrument used at a white heat. When applied at a white heat, and when the contact is made firmly and rapidly, there is scarcely any pain. Indeed, this very morning, a gentleman in this city, after being burned, was surprised to find that all was done, and was inclined to think I had merely tried to make him believe that it had been done. The application, in fact, is exceedingly easy. So slightly painful is it, that I have never seen a patient refuse it a second time after first receiving it. Among the means of counter irritation which may change the activity of the cells, these are certainly among the best, and in harmony with the theories I have put forward in this room. If we wish to change the morbid activity of the cells, no better means exist than applying irritation to the nerves of the skin in the parts which receive the nerves from the seat of the disease. But, as I believe that one side of the base of the brain has nerve fibers going to the two sides of the body, you may understand that I do not think it necessary to apply it to the paralyzed limbs. You may obtain success in applying it on the other side, as there is communication by the nerve fibers with the cells at the base of the brain, between the two sides of the body and the one side where the disease may exist.

But there are other means, and in the future when we pay more attention to what is taught by the power of arrest of activity, the treatment of disease of the brain will have gained immensely. If we examine what remedies may have power in cases of paralysis, we find that the most powerful is that one which we know to be able to increase the reflex excitability of the spinal cord at the base of the brain. Suppose the cells which move the various parts of the body to be stopped in their activity. Suppose the inhibition has taken place, as I believe, when there is paralysis. In such case, according to the theory, the cells of grey matter in the spinal cord as well as in the base of the brain itself, have their activity arrested. The best plan, theoretically as well as practically, is to make use of those internal means that can increase the reflex excitability; and nothing is as good as strychnia for that purpose. But as regards the dose it must be such as to produce a decided effect. When, in 1814, Fouquier made the first use of nux vomica, when he studied its action in paralysis, he invariably gave doses which were able to produce stiffness; and he obtained a most wonderful success. He was a most reliable man, a professor in a school of medicine in Paris and a physician to Louis Phillipe, and he certainly deserved his high position for his accuracy in observing facts. Since that time two or three medical men have practiced the use of strychnia in cases of paralysis caused by disease of the base of the brain. Fouquier gave doses, which, for a long time, kept his patients in a state of almost constant stiffness. There is no danger in producing that stiffness. The patient may be frightened, but if he have confidence in you, you can give it without risk. I have placed my own person under the influence of strychnia in such cases for days, without any other unpleasant sensation except inability to move about for a time. There is no doubt whatever that strychnia, in large doses, has a very great power indeed in increasing the reflex faculty of the spinal cord against paralysis, in cases of disease of the base of the brain. As regards the dose, it is a question of constitution. So begin with a small dose—one thirty-second of a grain three times a day. Go on increasing the dose gradually. I have seen patients bear perfectly well two-thirds, or even three-fourths of a grain in a day. But these patients are rare. Generally stiffness appears when the dose rises to one-tenth of a grain, three times a day. It is essential, if the dose is not sufficient, to go on increasing it cautiously. But you must be exceedingly careful, on account of the difference of dose necessary to produce a given result in different patients. In one case one-eighth of a grain may produce no effect, and yet a difference sufficient to increase the dose to one-seventh of a grain might produce an effect.

Therefore, the best plan is to dissolve a certain amount of strychnia in a large quantity of water, and go on day after day giving each day half a teaspoonful more. The measure must be accurate as otherwise there would be risk of producing too great effect. When you reach a dose producing stiffness, then go on measuring the same dose every day until the symptoms disappear. In some individuals such dose will be taken without effect; but it is exceedingly rare. There is no loss of power of the remedy and no increase. The old notion that strychnia increased in the system is in opposition to the facts. It is owing to the fact that, as I said, a moment ago, the addition of a minute quantity to a dose that produces no effect, will produce an effect. This has misled some persons. But I should like those among you who feel inclined to increase the dose, and who find that it can be brought up to two-thirds of a grain a day without producing convulsions, I should like those who might witness such a thing to be aware of the possibility that strychnia may not be as rapidly absorbed in the bowels of one person as in another. I had a patient who was taking nearly one grain a day without any special manifestation. I became a little nervous, and asked that the stools be sent to me. An examination was made by washing the stools with water, and then a frog was dipped in the water which had taken away simply a part of the strychnia. I found the frog to become tetanic, showing that there was strychnia in the stools. It proved that the absorption of the strychnia had not taken place in that case.

You may easily understand that a change might take place in such an individual, and after giving the strychnia for a time without any visible effect, suddenly there might occur symptoms which might frighten the patient, the family and perhaps yourself. The dose might be such as would endanger the life of the patient. It is, therefore, quite essential to test the stools, and ascertain whether or not the strychnia is absorbed.

I should like to dwell, gentlemen, much longer on this subject, but I regret that I must close. I have, therefore, only to thank the gentlemen who have been willing to listen to these disconnected sentences. I much regret, that by the necessity of curtailing, I have been obliged to pass important parts of the subject and to give assertions rather than demonstrations. But if, as I have some hope, the views that I have suggested are taken up by another man in the audience, I hope some good will come from the lectures. I have to thank you for your kind attention.

---

WE note with pleasure in the *American Journal of Obstetrics* for April, 1878, a communication from the pen of the senior editor of the CHICAGO MEDICAL JOURNAL AND EXAMINER. It relates to the question of the veracity of Dr. J. M. Rose, of West Winfield, N. Y., whose article entitled, "Four Successive Ruptures of the Uterus in the Same Patient," was first published by us in August of the year 1877. This paper has been extensively reproduced in Germany, France, Italy and Spain; generally with a protest against its acceptance as true.

Dr. Byford completely vindicates the reputation of our contributor, as a veracious and conscientious member of the profession.

J. N. H.

LONG NAILS IN THE KINGDOM OF ANNAM.—According to Lieutenant de Corbigny, literary men in Annam are known by the dimensions of their nails, which sometimes attain a length of twenty-five centimetres. The nail of one finger, however, is cut short, to enable its possessor to scratch himself—a laborious undertaking in all classes of that country. (*Le Tour du Monde*, Jan., 1878.)

